# Assisted injection among people who inject drugs in Thailand

**DOI:** 10.1186/1747-597X-8-32

**Published:** 2013-09-10

**Authors:** William K Lee, Lianping Ti, Kanna Hayashi, Karyn Kaplan, Paisan Suwannawong, Evan Wood, Thomas Kerr

**Affiliations:** 1 British Columbia Centre for Excellence in HIV/AIDS, St. Paul’s Hospital, 608-1081 Burrard Street, V6Z 1Y6, Vancouver, BC, Canada; 2 Department of Medicine, University of British Columbia, 2775 Laurel Street, 10th Floor, V5Z 1M9, Vancouver, BC, Canada; 3 Thai AIDS Treatment Action Group, 18/89 Vipawadee Road, soi 40 Chatuchak, Bangkok 10900, Thailand

**Keywords:** Injection drug use, Thailand, Assisted injection

## Abstract

**Background:**

Assisted injection is common among people who inject drugs (IDU), and has been associated with elevated risk for HIV infection and overdose. However, this practice has not been explored in the Asian context, including in Thailand, where HIV prevalence among IDU remains high.

**Methods:**

Using multivariate logistic regression, we examined the prevalence and correlates of assisted injecting among IDU participating in the Mitsampan Community Research Project in Bangkok. We also sought to identify reasons for engaging in assisted injecting and those who provide this form of assistance.

**Results:**

In total, 430 IDU participated in this study, including 376 (87.5%) who reported having ever required assistance injecting, and 81 (18.8%) who reported assisted injecting in the previous six months. In multivariate analyses, assisted injecting in the previous six months was independently and positively associated with being female (adjusted odds ratio [AOR] = 2.42; 95% confidence interval [CI]: 1.40 – 4.18), being a weekly heroin injector (AOR = 1.78; 95% CI: 0.99 – 3.20), syringe sharing (AOR = 2.08; 95% CI: 1.18 – 3.68) and soft-tissue infection (AOR = 3.51; 95% CI: 1.43 – 2.53). Having a longer injecting career (AOR = 0.96; 95% CI: 0.94 – 0.99) was negatively associated with assisted injecting. Primary reasons given for engaging in assisted injecting included being new to injecting and lacking knowledge on how to inject. The most common providers of assistance with injecting were close friends.

**Conclusion:**

We found a high prevalence of assisted injecting among IDU in Bangkok, with females, frequent heroin injectors, those with shorter injecting careers being more likely to engage in this practice. Those who require help with the injecting process are more likely to share syringes, and have skin infections. These findings indicate the need for interventions focused on promoting safer and self-administered injections.

## Introduction

The injection of illicit drugs remains an international public health concern and has been associated with the transmission of the human immunodeficiency virus (HIV) and other serious health-related problems [1,2]. As such, various harm reduction strategies, including needle distribution programs, have been implemented to mitigate high-risk behaviors such as syringe sharing, which contribute to the spread of blood-borne diseases [3-6]. Despite these measures, people who inject drugs (IDU) continue to be exposed to a range of drug-related harms [7].

In North American settings, the provision of manual assistance with injections among people who inject drugs (IDU) has received increasing attention, as it has been demonstrated to be independently associated with elevated risk for blood-borne disease transmission, infections, non-fatal overdose and other health-related problems [4,8]. One study indicated that syringe sharing – a behavior strongly associated with HIV transmission – is four times more likely to occur among those who receive help with drug injections than regular IDU, as the “street doctors” (injectors) are likely to reuse a needle that they have already used on themselves when injecting others [4]. Furthermore, Kral et al. found that the use of blood-contaminated materials such as used cottons or thumbs to clean the injection site is more commonly observed among those who receive help with their injections compared to those who self-administer their injections in the San Francisco Bay area [4]. Such a behavior allows for possible routes of HCV (Hepatitis C Virus) and possibly HIV transmission to occur. Skin-popping (i.e., injecting subcutaneously or intramuscularly) was also more likely to occur among IDU who receive help with their injections. This behavior has been associated with a higher risk of acquiring skin infections such as abscesses and necrotizing fasciitis [9]. The practice of assisted injecting has been found to be more common among women, those who are newer to injecting, and frequent cocaine injectors [8,10]. Several reasons for requiring help injecting have been identified and include perceived loss of accessible veins, difficulty with injecting because of shaky hands, and lack of familiarity with injection techniques [8].

Although assisted injecting has been identified in various settings as being a high-risk behavior, this practice has not been explored in the Asian context, including in Thailand where HIV prevalence among IDU has remained persistently high [11]. In an effort to inform related public health responses, this study aims to identify the prevalence and correlates of assisted injecting among IDU in Bangkok, Thailand. We also sought to identify reasons for needing assistance with injecting and those who typically provide this type of assistance.

## Methods

### Study design

Data for this study were derived from the Mitsampan Community Research Project, a collaborative research effort involving the Mitsampan Harm Reduction Center (MSHRC), a drug user–run drop-in centre in Bangkok, Thailand, the Thai AIDS Treatment Action Group (Bangkok, Thailand), Chulalongkorn University (Bangkok, Thailand), and the British Columbia Centre for Excellence in HIV/AIDS/University of British Columbia (Vancouver, Canada).

Between July and October of 2011, the research partners recruited and surveyed 440 IDU. Potential participants were recruited through peer outreach efforts and word-of-mouth, and were invited to attend the MSHRC or O-Zone House (another drop-in centre in Bangkok) in order to be part of the study. Adults residing in Bangkok or in adjacent provinces who had injected drug(s) in the past six months were eligible for participation. All participants provided informed consent and completed an interviewer-administered questionnaire eliciting a range of information, including socio-demographic characteristics, drug use patterns, and experiences with drug law enforcement and accessing healthcare. Peer researchers (i.e., current and former IDU) trained to conduct outreach were sent offsite in pairs to areas with a high density of IDU to recruit participants. Potential participants were given information cards with directions to the MSHRC or O-Zone House and further contact instruction. All participants provided informed consent and completed an interviewer-administered questionnaire eliciting a range of information, including socio-demographic characteristics, drug use patterns, and experiences with drug law enforcement and accessing healthcare. The questionnaire was administered by a group of peer researchers who underwent proper and extensive training by frontline staff from the BC Centre for Excellence in HIV/AIDS. Upon completion of the questionnaire, participants received a stipend of 350 Thai baht (approximately US$12). The study was approved by the research ethics boards at Chulalongkorn University and the University of British Columbia.

For the present analyses, the primary outcome of interest was reporting assisted injecting in the past six months by responding “always”; ”most of the time”; “sometimes” or “not very often” (as opposed to “never”) to the question: “In the last 6 months, how often did anyone help you inject?” We considered several potential explanatory variables of interest, including: gender (female vs. male); median age (≥ 38 years vs. < 38 years); education (≥ secondary education vs. < secondary education); relationship status (married or having a regular partner vs. other); heroin injection (> weekly vs. ≤ weekly vs. no injection), midazolam injection (> weekly vs. ≤ weekly vs. no injection); methamphetamine “yaba” injection (≥ weekly vs. ≤ weekly vs. no injection), crystal methamphetamine “ice” injection (≥ weekly vs. ≤ weekly vs. no injection), lent or borrowed syringes to/from others (yes vs. no); used drugs in combination (yes vs. no) length of injecting career (per year longer), non-fatal overdose (yes vs. no) and soft-tissue infections (yes vs. no). All variables referred to the previous six months unless otherwise indicated. The covariates in our study were chosen based on a number of studies in the North American context that suggest significant and independent associations with assisted injecting [10].

Bivariate statistics and multivariate logistic regression were applied to identify factors associated with assisted injecting. Categorical explanatory variables were analyzed using Pearson’s Chi-square test and Fisher’s exact test (when one or more cells contained values less than or equal to five), and continuous variables were analyzed using simple logistic regression. We then applied an *a priori*-defined statistical protocol based on examination of the Akaike Information Criterion (AIC) and *p*-values to construct an explanatory multivariate logistic regression model. As a first step, we constructed a model including all variables significantly associated with the outcome at *p* ≤ 0.10 in bivariate analyses. After the AIC of the model was noted, subsequent variables with the largest p-value were individually removed and a reduced model was built. We continued this iterative process until a combination of variables with the lowest p-value yielded the lowest possible AIC value. All *p*-values were two-sided.

In secondary analyses, participants who ever reported requiring help injecting were asked why they needed help injecting. Furthermore, these participants were asked who provided them assistance with injecting. These data are presented using descriptive statistics.

## Results

In total, 430 IDU, including 83 (19.3%) females, provided complete data and were included in this analysis. The median age of participants was 38 years (interquartile range: 34 – 48 years). In total, 376 (87.4%) reported having ever required assistance injecting, while 81 (18.8%) participants reported that they had engaged in assisted injection in the last six months. Among those who have reported needing help injecting in the last 6 months, 12 (14.8%) said they always required help, 10 (12.3%) said they needed help most of the time, 25 (30.9%) required help some times, and 34 (42.0%) did not require assistance very often.

As shown in Table 1, in bivariate analyses, factors positively associated with assisted injecting at the 0.10 level included being female (odds ratio [OR] = 2.42; 95% confidence interval [CI]: 1.40 – 4.18), heroin injection of more than once a week (OR = 1.78; 95% CI: 0.99 – 3.20), being in a relationship (OR = 1.60; 95% CI: 0.98 – 2.59), shared syringes (OR = 2.08; 95% CI: 1.18 – 3.68), used drugs in combination (OR = 1.53; 95% CI: 0.92 – 2.53) and soft-tissue infections (OR = 3.51; 95% CI: 1.43 – 8.64). Factors negatively associated with assisted injecting included being older or equal to the age of 38 (OR = 0.52; 95% CI: 0.32 – 0.84), heroin injection once a week or less (OR = 0.61; 95% CI: 0.33 – 1.10) and having a longer injection career (OR = 0.96; 95% CI: 0.94 – 0.99).

**Table 1:** Bivariate analyses of factors associated with assisted injecting in the past six months among IDU in Bangkok, Thailand (n = 430)

	Required help injecting*			
**Characteristic**	**Yes 81 (18.8%)**	**No 349 (81.2%)**	**Odds ratio (95% CI)**	***p *- value**
**Median Age**				
≥ 38 years	32 (39.5)	195 (55.9)	0.51 (0.31 – 0.84)	0.008
< 38 years	49 (60.5)	154 (44.1)		
**Gender**				
Female	26 (32.1)	57 (16.3)	2.42 (1.40 – 4.18)	0.001
Male	55 (67.9)	292 (83.7)		
**Education**				
≥ Secondary education	51 (63.0)	212 (60.7)	1.10 (0.67 – 1.81)	0.712
< Secondary education	30 (37.0)	137 (39.3)		
**Relationship status**				
Married or having a regular partner	44 (54.3)	149 (42.7)	1.60 (0.98 – 2.59)	0.058
Other	37 (45.7)	200 (57.3)		
Heroin injection*				
>Weekly	27 (33.3)	64 (18.3)	1.78 (0.99 – 3.20)	0.054
≤Weekly	21 (25.9)	146 (41.8)	0.61 (0.33 – 1.10)	0.096
No injection	33 (40.7)	139 (39.8)		
Midazolam injection*				
>Weekly	42 (51.9)	195 (55.9)	0.92 (0.52 – 1.63)	0.776
≤Weekly	17 (21.0)	60 (17.2)	1.21 (0.59 – 2.46)	0.598
No injection	22 (27.2)	94 (26.9)		
Yaba injection*				
>Weekly	16 (19.8)	72 (20.6)	1.08 (0.57 – 2.04)	0.818
≤Weekly	25 (30.9)	83 (23.8)	1.46 (0.83 – 2.56)	0.185
No injection	40 (49.4)	194 (55.6)		
Ice injection*				
>Weekly	2 (2.5)	17 (4.9)	0.50 (0.11 – 2.21)	0.546
≤Weekly	10 (12.3)	39 (11.2)	1.09 (0.52 – 2.29)	0.847
No injection	69 (85.2)	293 (84.0)		
Shared syringes*				
Yes	22 (27.2)	53 (15.2)	2.08 (1.18 – 3.68)	0.011
No	59 (72.8)	296 (84.8)		
**Number of years injecting**				
Median (IQR)	17 (8 – 21)	19 (15 – 27)	0.96 (0.94 – 0.99)	0.003
Non-fatal overdose*				
Yes	4 (4.9)	11 (3.2)	1.60 (0.50 – 5.15)	0.498
No	77 (95.1)	338 (96.8)		
Used drugs in combination*				
Yes	53 (65.4)	193 (55.3)	1.53 (0.92 – 2.53)	0.097
No	28 (34.6)	156 (44.7)		
Soft-tissue infections*				
Yes	9 (11.1)	12 (3.4)	3.51 (1.43 – 8.64)	0.004
No	72 (88.9)	337 (96.6)		

*IDU* people who inject drugs, *CI* confidence interval, *IQR* interquartile range.

*Activities/behaviors in the previous six months.

As indicated in Table 2, in multivariate analyses, assisted injecting remained independently associated with being female (AOR = 2.45; 95% CI: 1.33 – 4.48), being a frequent heroin injector (AOR = 1.41; 95% CI: 1.01 – 1.98), syringe sharing (AOR = 2.17; 95% CI: 1.18 – 3.94) and soft-tissue infections (AOR = 3.02; 95% CI: 1.14 – 7.72). Having a longer injecting career (AOR = 0.97; 95% CI: 0.94 – 0.99) remained negatively associated with assisted injecting.

**Table 2:** Multiple logistic regression of factors associated with assisted injecting among IDU in Bangkok, Thailand (n = 431)

**Variable**	**Adjusted odds ratio (AOR)**	**95% confidence interval (CI)**	***p *- value**
**Gender**			
(Female vs. Male)	2.45	(1.33 – 4.48)	0.004
Heroin injection*			
(>Weekly vs. **≤**Weekly vs. No injection)	1.41	(1.01 – 1.98)	0.043
**Number of years injecting**			
(Per year longer)	0.97	(0.94 – 0.99)	0.022
Shared syringes*			
(Yes vs. No)	2.17	(1.18 – 3.94)	0.011
Soft-tissue infections*			
(Yes vs. No)	3.02	(1.14 – 7.72)	0.022

*IDU* people who inject drugs.

*Activities/behaviors in the previous six months.

The two most common self-reported reasons for requiring assistance with injecting include being new to injecting (68.7%) and not knowing how to inject (56.1%), and 21.8% attributed bad veins as being a reason for why they needed help injecting. Less common reasons for requiring assistance injecting include requiring groin injection (9.7%), having shaky hands (5.8%), being drug-sick (4.1%), and hating needles (3.4%). Finally, of the participants who ever needed assistance with injecting, 327 (84.9%) received assistance from a close friend, 42 (10.9%) reported receiving help from a regular partner, followed by 33 (8.6%) who received assistance from an acquaintance.

## Discussion

In the present analysis, we found that approximately 88% of a community-recruited sample of IDU in Bangkok had reported previously needing assistance to inject drugs, and 19% reporting receiving assistance with an injection in the past six months. In multivariate analyses, we found five variables that remained positively and independently associated with assisted injecting. Soft-tissue infection was the variable most strongly associated with the outcome, with people who have soft-tissue infections being approximately three times more likely to need assistance with injecting. Those who are female and those who have shared syringes were both found to be more than twice as likely to engage in assisted injecting. Frequent heroin injectors were about 1.5 times more likely to require help with injecting. Lastly, having a longer injecting career was slightly negatively associated with assisted injecting. The most common reasons given for requiring assisted injecting are not knowing how to inject and being new to injecting.

Despite the fact that this is, to our knowledge, the first study to explore the practice of assisted injecting in Thailand, some of our findings are consistent with a large body of literature on assisted injecting in North American settings [6,8,10]. For instance, we found that female IDU were approximately twice as likely to engage in assisted injecting compared to male IDU. This may be partly explained by the gender dynamics common among IDU populations, whereby men often retain control over the possession and administration of drugs [12-14]. Accordingly, women are often injected by male friends [5], and as a consequence often do not learn how to self-administer their injections. In a study conducted by Fairbairn and colleagues in Canada, women gave narrative accounts of assisted injecting, detailing the opportunity to share the injecting process and drug high with men, hence fostering an increased sense of trust and intimacy [15]. Another reason that females in the Thai context might be more than twice as likely to engage in assisted injecting than males is because females naturally have smaller veins and may not know how to inject themselves, as has been shown in other settings [5]. Although many other studies have shown the importance of gender dynamics in influencing the injection process among women, it is important to note that being in a relationship, a status sometimes associated with trust and intimacy, was not independently associated with assisted injecting in our study. This inconsistency further highlights the need to explore gender dynamics, outside of intimate relationships, and the impact of such dynamics on assisted injecting among female IDU in Thailand.

Our study also found that more experienced IDU were less likely to require help injecting. These findings are further reflected in our sub-analyses where the most common reasons given for needing assistance with injecting were being new to injecting and not knowing how to inject. Our multivariate analysis also supports this finding by demonstrating that IDU who had shorter injection careers were more likely to require assistance with injection. These results are consistent with O’Connell et al.’s [10] study on assisted injecting as a predictor for HIV infection among IDU in Vancouver which found that younger IDU and more recent initiates to injecting were more at risk to requiring assistance injecting. Furthermore, Wood et al.’s [6] findings on Vancouver IDU mirror our results, in that 7% of men and 13% of women in that study attributed requiring assistance with injecting to not knowing how to inject properly.

Our study found that frequent heroin injectors were more likely to engage in assisted injecting compared to less frequent heroin injectors. However, research in North America points to frequent stimulant use (cocaine use in particular) as being more strongly associated with assisted injecting [8,16]. On the other hand, previous research has also identified withdrawal effects (i.e., “drug sickness”) as a reason for assisted injecting [6], and this type of withdrawal is more commonly associated with heroin injecting. This geographical variance observed herein could possibly be accounted for by the fact that there are different drug use patterns across different geographical regions. For example, it is well known that cocaine injecting is virtually non-existent among Thai IDU [17].

Our findings indicate that those who are recipients of injections are more likely to have soft-tissue infections such as abscesses. Several publications have stated that a strong risk factor for skin infections is skin-popping, where the injection is intramuscular or subcutaneous [4,18]. Although intravenous injection carries its own risks, intramuscular injections allow infections to occur because foreign substances are introduced directly into the tissue where it remains localized and concentrated, providing it with an opportunity to fester and infect the skin. It is possible that skin-popping is more commonly observed among Thai IDU who are recipients of injections because those who administer the injection may not know how to inject properly themselves. This phenomenon could be social in origin, where younger and novice injectors tend to use drugs with their friends who coincidentally would be closer in age to each other and have similar drug-use experience.

One of the greater concerns of assisted injecting is the loss of control of the injection process, which can lead to risky behaviors such as syringe sharing and the over-administration of a drug, which can lead to overdose [8,19]. Although syringe sharing among Thai IDU was strongly associated with having engaged in assisted injecting, non-fatal overdose was not significantly associated with requiring help injecting, which runs counter to the findings from the North America context [8]. Accordingly, more research is needed to identify the impact of assisted injecting on overdose risk among Thai IDU.

Collectively, our findings highlight the need for interventions that reduce the practice of receiving assisted injecting among Thai IDU. For this reason, based on our findings that syringe sharing is independently associated with assisted injection among Thai IDU, it would be beneficial to encourage the wide implementation of such programs in the Thai context. However, based on our current knowledge and the present findings [6,8], the provision of sterile injection equipment alone would do little to avert the practice of assisted injecting altogether. In addition to the provision of clean equipment and education on safe injection, other harm reduction strategies such as supervised injection facilities (SIFs) may be helpful in both reducing the harmful effects that accompany assisted injecting, such as HIV transmission, overdose, public drug use, and reducing the prevalence of this practice altogether [20-23]. Evaluations have found that staff within SIFs often provide education on safer injection techniques, and increases in safer injection practices among high-risk IDU have been associated with exposure to SIFs [8].

However, it should be noted that at most SIFs, assistance provided to IDU who need assistance with injection is limited to verbal directions and minimal manual assistance (excluding the actual act of injection). Some IDU are able to conduct the injection successfully following the receipt of such support, but there still remain a number who are unable to properly inject, and consequently seek out other IDU to assist with the injection [16]. Therefore, the policies and rules surrounding assisted injection in SIFs deserve further investigation [24]. In addition, given that SIFs have not yet been implemented at all in low- and middle-income settings, a site assessment in Bangkok on the feasibility and effectiveness of operating a SIF should be conducted first. For example, the IDU population in Bangkok may be too dispersed geographically, and therefore a micro-environmental intervention of this kind may not reach high levels of coverage [25]. Further, a lack of key stakeholder support may serve to undermine the effectiveness of such an approach.

Female and novice IDU attributed requiring help injecting to not knowing how to inject properly. With the provision of appropriate educational support, this specific group of IDU can benefit from learning proper injecting. For those IDU who continue to receive assistance with injections, they may be able to learn about the deleterious effects of using previously used equipment, including the elevated risk of disease transmission. Such educational preventative measures have been documented in the North American context and have been shown to be effective [26,27]. However, it should be noted that the current trend is that messages of safe injection assume self-administered injections, thus future educational campaigns could shift their focus and be aimed at those who specifically receive or deliver injections. Given that drug use remains heavily criminalized in Thailand, and IDU experience many social barriers to healthcare, peer-based educational programming may be most effective in addressing the problem of assisted injecting in this setting.

This study has limitations. First, the study sample was not randomly selected and therefore may not be representative of all local IDU. Hence, this study may not be generalizable to Thai IDU or IDU in other settings. However, given that no accessible official registries of IDU exist in this setting, deriving a random sample was not possible. Second, the study relied on self-reported data, which may be subject to response biases. Third, the study was cross-sectional in nature, and therefore we were unable to determine temporal relationships between the outcome and explanatory variables considered.

In conclusion, we found that approximately 88% of IDU in Bangkok reported a history of assisted injecting, and 18% reported receiving assistance with injecting in the previous six months. Those engaging in assisted injecting were more likely to be female and frequent heroin injectors. Those with longer injecting careers were less likely to report assisted injecting. A lack of knowledge of how to inject was the most common reason given for engaging in the practice. Assisted injecting among this population was also strongly associated with syringe sharing and soft-tissue infections. These findings point to the need for program development within Thailand to reduce the risks and health consequences associated with assisted injecting. More specifically, efforts should be made to widely implement educational and peer-based interventions focused on safer injecting in this setting.

## Competing interests

The authors declare that they have no competing interests.

## Authors’ contributions

The specific contributions of each author are as follows: WL, LT and TK were responsible for the research design; LT conducted the statistical analyses; WL prepared the first draft of the manuscript; All authors provided critical comments on the first draft of the manuscript and approved the final version to be submitted.
